# Immunogenicity and protective efficacy of recombinant adenovirus expressing a novel genotype G2b PEDV spike protein in protecting newborn piglets against PEDV

**DOI:** 10.1128/spectrum.02403-23

**Published:** 2023-12-04

**Authors:** Xin Song, Qun Zhou, Jiaqi Zhang, Taoyun Chen, Gunan Deng, Hua Yue, Cheng Tang, Xuejing Wu, Jifeng Yu, Bin Zhang

**Affiliations:** 1 College of Animal & Veterinary Sciences, Southwest Minzu University, Chengdu, China; 2 Key Laboratory of Ministry of Education and Sichuan Province for Qinghai-Tibetan Plateau Animal Genetic Resource Reservation and Utilization, Chengdu, China; 3 Sichuan Provincial Key Laboratory of Animal Breeding and Genetics, Sichuan Animal Science Academy, Chengdu, China; David Geffen School of Medicine at UCLA, Los Angeles, California, USA

**Keywords:** porcine epidemic diarrhea virus, G2b genotype, S protein, adenoviral vector, immune efficacy, protective efficacy

## Abstract

Porcine epidemic diarrhea virus (PEDV) causes porcine epidemic diarrhea (PED). The emergence of the novel G2b strains of PEDV has increased the need for new vaccines. We generated a recombinant adenovirus expressing the spike (S) protein of G2b PEDV based on human adenovirus serotype 5 (Ad5), named rAd5-PEDV-S. Immunization with rAd5-PEDV-S elicited a significant PEDV-specific humoral immune response in sows, including IgA and IgG in colostrum and serum and circulating neutralizing antibodies. The efficacy of rAd5-PEDV-S was superior to that of the commercial inactivated vaccine, and the intramuscular (IM) route was more effective than the intranasal route. Interestingly, there was no significant difference in immunization effect between the twice and once-immunized IM groups (administered 5 weeks before farrowing). Five-day-old piglets born to sows immunized with the rAd5-PEDV-S through the IM route had less diarrhea and weight loss when challenged with PEDV. Fecal PEDV RNA expression in the PEDV-challenged rAd5-PEDV-S IM group piglets was considerably lower than that in the other groups; all five piglets from the rAd5-PEDV-S IM group survived the infection period. Histopathological examination of small intestinal sections revealed evident shedding of ileum intestinal mucosal epithelial cells in the phosphate-buffered saline (PBS) group. These findings suggest that vaccinating pregnant sows with rAd5-PEDV-S induces immune responses in the pregnant sows and passively protects piglets. Our findings highlight the potential of rAd5-PEDV-S as a candidate vaccine for PED.

**IMPORTANCE:**

Porcine epidemic diarrhea (PED) is a highly infectious and economically significant gastrointestinal disorder that affects pigs of all ages. Preventing and controlling PED is achieved by immunizing sows with vaccines, enabling passive piglet immunization via colostrum. The prevalence of G2b porcine epidemic diarrhea virus (PEDV) continues in China despite the use of commercial vaccines, raising questions regarding current vaccine efficacy and the need for novel vaccine development. Adenovirus serotype 5 (Ad5) has several advantages, including high transduction efficiency, a wide range of host cells, and the ability to infect cells at various stages. In this study, we expressed the immunogenic proteins of spike (S) using an Ad5 vector and generated a PED vaccine candidate by inducing significant humoral immunity. The rAd5-PEDV-S prevented PED-induced weight loss, diarrhea, and intestinal damage in piglets. This novel vaccine candidate strain possesses the potential for use in the pig breeding industry.

## INTRODUCTION

Porcine epidemic diarrhea virus (PEDV) is responsible for porcine epidemic diarrhea (PED), an acute, lethal, and highly contagious disease in wild and domestic pigs (1). PEDV was first detected in Europe and has since spread widely, evolving to form mutant strains with substantial virulence and infectivity, causing significant losses to the global swine industry (2). The prevalent PEDV strains are classified into two genotypes, G1 (classical) and G2 (variant), of which the G1 genotype includes two subtypes, G1a and G1b, and the G2 genotype includes three subtypes, G2a, G2b, and G2c (3). The G2b PEDV strains are highly virulent non-S-INDEL strains recombined within the spike (S) protein domain, which first emerged in 2011–2012. The prevalence of G2b PEDV caused devastating disease and substantial economic losses (4 – 6).

PEDV is an enveloped single-strand positive-sense RNA virus in the family *Coronaviridae*, genus *Alphacoronavirus* (7). The PEDV genome is approximately 28 kb in length and comprises a 5´ untranslated region (UTR), at least seven open reading frames (ORFs), and 3´ UTR (7). ORF1a and ORF1b encode two replicase polyproteins, which are later proteolytically processed into various non-structural proteins. The remaining ORFs in the 3´ terminal region encode five structural proteins: S, envelope, membrane, nucleocapsid proteins, and accessory protein, ORF3 (7). The S protein of coronaviruses is responsible for the induction of neutralizing antibodies (NAb), specific receptor binding, and cell membrane fusion (8, 9). Therefore, the S protein as the immunogen has been considered for developing novel vaccines (8, 10).

Vaccination is the best way to prevent PEDV infection at a very early stage of infection by transferring maternal antibodies to piglets through the vaccination of sows (11, 12). Several live attenuated and inactivated/killed vaccines have been developed and are commercially available in many countries (13, 14). Same as other coronaviruses, the S gene of PEDV has shown significant variation. Previous study showed that the S proteins of G1 strains and G2 strains differed greatly from each other, with a similarity rate of only 90–96% (2). Due to the current genetic variation of PEDV, the effectiveness of existing vaccines faces challenges (13, 15). Commercial vaccines cannot completely block suckling piglets from being infected by PEDV. Inactivated vaccines are safe but have a short duration of immunity and require the appropriate adjuvants for strong immune responses. Live-attenuated vaccines, produced by serially passaging field strains, are more effective against homologous strains but have a long lead development time and cannot supply enough protection to highly virulent heterologous strains (16). The existing PED commercial vaccines do not provide cross-protection, and the same poor cross-protection situation has been observed in other coronaviruses. The emergence of a tremendous number of mutant severe acute respiratory syndrome (SARS-CoV-2) strains (more than eight million) has reduced the protection afforded by existing vaccines, resulting in breakthrough infection (17). Vaccines with improved efficacy are urgently needed to control PEDV infections effectively. Human adenovirus serotype 5 (Ad5) has several advantages, including high transduction efficiency, a wide range of host cells, and the ability to infect cells at various stages (9). Ad5 has been applied in human and animal vaccine research for many years (9, 18, 19) and has been successfully used to prevent and control many infectious diseases; it has also been confirmed to be safe and effective (9, 20 – 22).

This study aimed to develop a vaccine against PED using a recombinant adenovirus based on Ad5 to express the S protein. The potential immune responses of this vaccine were evaluated in sows, and a challenging experiment was conducted in newborn piglets to determine efficacy after vaccination. These findings provide a foundation for research in developing a vaccine against PED.

## RESULTS

### Construction and identification of rAd5-PEDV-S

**Fig 1:**
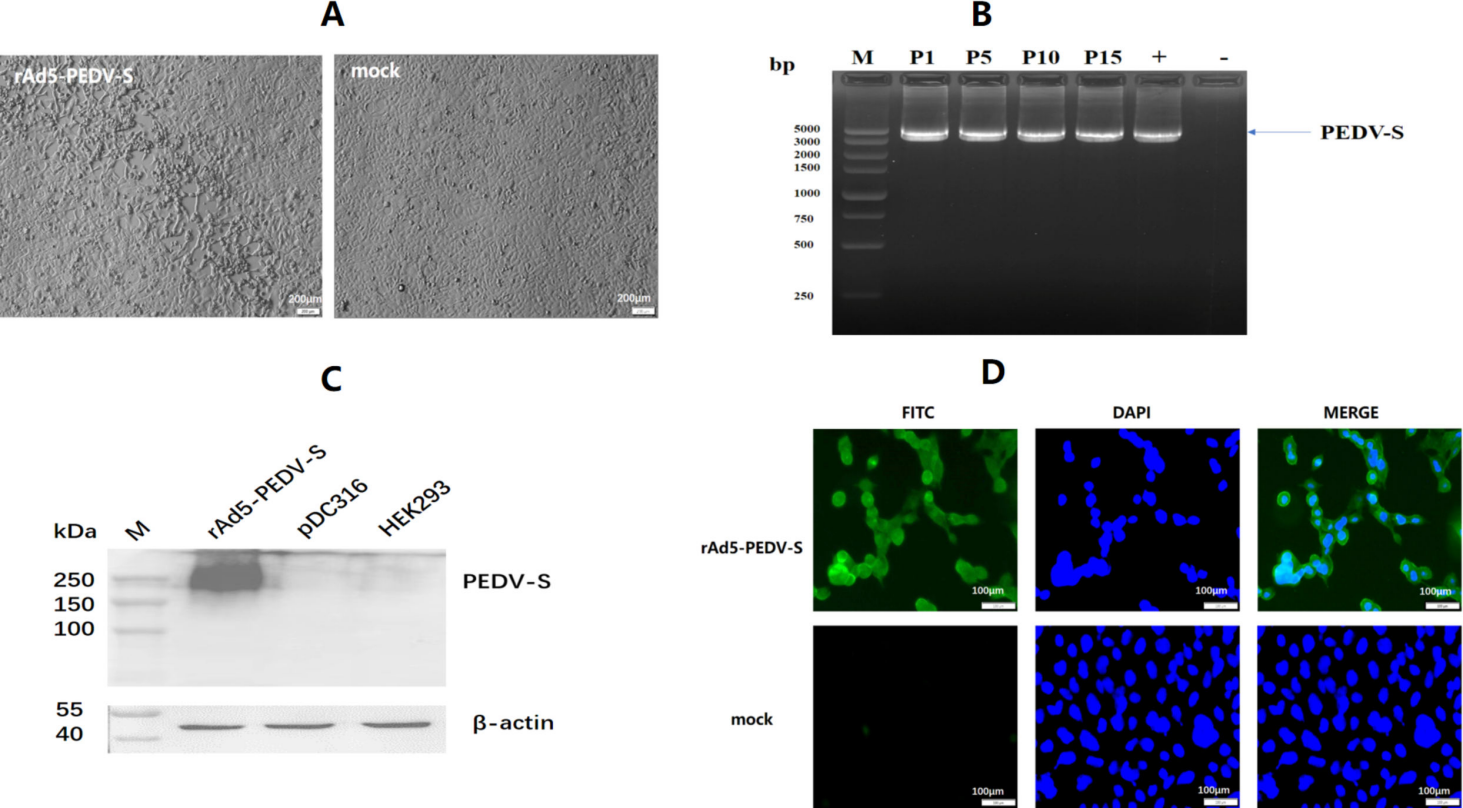
Construction and identification of rAd5-PEDV-S. (**A**) Ad-related cytopathic effect (CPE) caused by rAd5-PEDV-S in HEK293 cells. The left figure represents CPE caused by rAd5-PEDV-S, right represents normal cells. (**B**) The genetic stability of rAd5-PEDV-S in HEK293 cells was detected by PCR, “P” means passage. “+” means positive control, “-” means negative control. (**C**) Western blotting analysis of S protein expressed by rAd5-PEDV-S in HEK293 cells. (**D**)Immunofluorescence assay (IFA) analysis of S protein expressed by rAd5-PEDV-S in HEK293 cells.

The full-length S gene of novel G2b PEDV variant CH/TP-4-4/2018 (GenBank accession number: MK140814.1) was selected as the immunogen; it has approximately 96.61%–100% amino acid homology with PEDV strains collected in recent years (23). The length of the complete coding sequence was 4,158 bp, corresponding to 1,386 aa. The S gene was optimized for increased antigen expression in human embryonic kidney 293 (HEK293) cells; the guannine and cytosine (GC) content-optimized S gene improved from 41.1% to 52.2%. The codon adaptation index was 0.67 to 0.85 (0.8–1.0 was considered to favor protein expression) (9, 24). The recombinant adenovirus (rAd5-PEDV-S) was successfully packaged in HEK293 cells through homologous recombination, resulting in typical cytopathic effects related to adenoviruses forming on the cells (Fig. 1A). PCR analysis of serially passaged rAd5-PEDV-S showed that the S gene was detectable at every passage, and sequencing confirmed that the result was accurate (Fig. 1B), indicating genetic stability of the recombinant adenovirus.

The expression of the S protein was successfully verified by western blotting (Fig. 1C). The expression of the S protein was also successfully verified by IFA, as specific green fluorescence was observed under fluorescence microscopy (Fig. 1D). These results demonstrated that rAd5-PEDV-S effectively expressed the S protein in HEK293 cells.

### rAd5-PEDV-S purified by chromatography

To improve viral purity, rAd5-PEDV-S was purified using chromatography on a Diamond Layer 700 BA (which utilizes anionic and hydrophobic interactions and molecular sieving) and a Diamond Q Mustang (which employs ion exchange action) for chromatographic purification. The resulting purification product’s optical density ratio (OD260/OD280) was 1.28, indicating the purity met the requirements. The expression of S protein in HEK293 cells infected with purified rAd5-PEDV-S was confirmed using western blotting. The tissue culture infective dose of 50% (TCID_50_) was calculated using the Reed-Muench method and was 1 × 10^8.24^/0.1 mL. The total number of virus particles (VPs) was 1.8 × 10^11^ VP/mL. These results demonstrate that the rAd5-PEDV-S was successfully purified.

### rAd5-PEDV-S induces PEDV-specific IgA and IgG antibodies in sows

**Fig 2:**
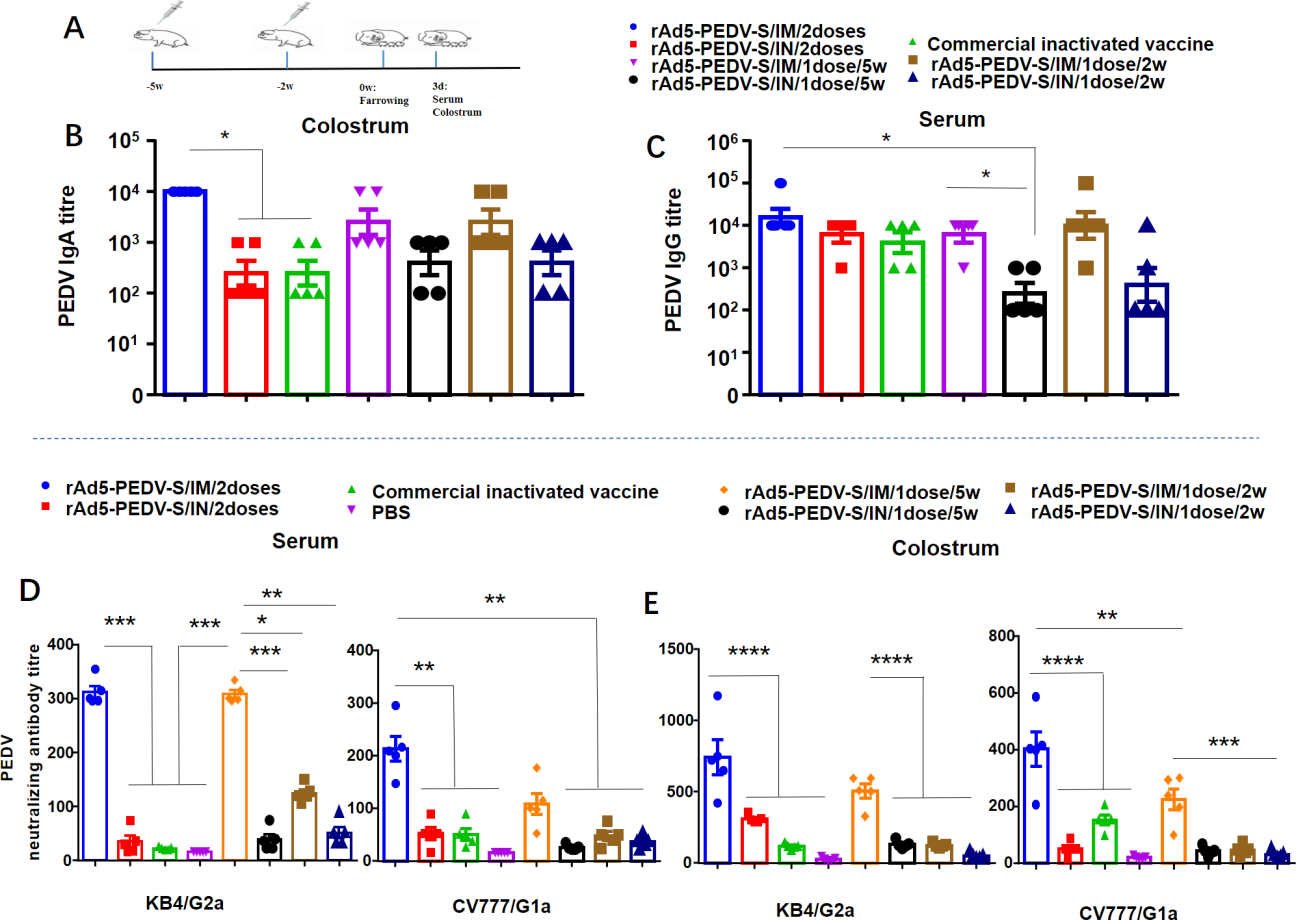
Humoral immune responses of rAd5-PEDV-S in sows. (**A**) Schematic representation of the immunization procedure. These sows being immunized with a primary immunization at 5 weeks before farrowing and a secondary immunization with the same dose irradiated 2 weeks before farrowing and immunized only once each at 5 weeks before or 2 weeks before farrowing. (**B**) IgA antibody titers in colostrum. (**C**) IgG antibody titers in serum. (**D**) NAb titers in colostrum assay in Vero cells. (**E**) NAb titers in serum assay in Vero cells. (Data were analyzed by Tukey’s multiple comparison test for subsequent analysis. Each point represents the titer of an individual. *, *P* < 0.05; **, *P* < 0.01; ***, *P* < 0.001; ****, *P* < 0.0001; ns, no significance).

To evaluate the humoral immune responses to rAd5-PEDV-S, we collected colostrum and serum samples from immunized sows on the third day postpartum to measure IgA and IgG (Fig. 2A). There were no adverse effects or abortions after immunization in any of the sows, and the litter size was not significantly different from that of the unimmunized group (Fig. S1).

An indirect enzyme-linked immunosorbent assay (ELISA) was employed to determine the PEDV-specific IgG and IgA antibody titers in immune sows’ serum and colostrum samples. The IgA titers for the twice-immunized rAd5-PEDV-S group administered through the intramuscular (IM) route were up to 1:10^4.0^, and the IgA titers for the rAd5-PEDV-S IM group were significantly higher than those in the intranasal (IN) group or the commercial inactivated group (Fig. 2B; Table S1). We also measured the IgG titers in different vaccination groups and found that the twice-immunized rAd5-PEDV-S IM group had IgG titers up to 1:10^4.3^. There were no significant differences in the IgG titers between the rAd5-PEDV-S IM group, rAd5-PEDV-S IN group, and the commercial inactivated vaccine group (*P* > 0.05) (Fig. 2C; Table S2).

Therefore, two doses of IM administration of rAd5-PEDV-S led to significantly higher levels of IgA response but just showed a slight elevation in IgG levels compared to unvaccinated sows. Interestingly, the twice-immunized rAd5-PEDV-S IM group was higher than the once-immunized IM group (administered at 5 weeks before farrowing), but there were no significant differences in humoral immune response between the twice immunized rAd5-PEDV-S IM group and the once-immunized IM group (administered at 5 weeks before farrowing) (*P* > 0.05). These findings suggest that the rAd5-PEDV-S vaccine effectively induces high levels of humoral immune responses in sows.

### rAd5-PEDV-S induces PEDV NAb in sows

To assess the humoral immune responses to rAd5-PEDV-S, samples of colostrum and serum were collected from immunized sows on the third day postpartum to detect NAb. A virus neutralization test determined the presence of NAb against PEDV in colostrum and serum samples.

In the twice-immunized rAd5-PEDV-S IM group, the NAb titers in the colostrum were 1:748 (KB4 strain) and 1:404 (CV777 strain), while in the serum, the titers were 1:315 or 1:216, significantly higher than those in the IN group and the commercial inactivated vaccine group (Fig. 2D and E; Tables S3 and S4). Interestingly, there was no significant difference between the twice-immunized rAd5-PEDV-S IM group and the once-immunized IM group (administered at 5 weeks before farrowing) (*P* > 0.05) (Fig. 2D and E; Tables S3 and S4). These results suggest that rAd5-PEDV-S induces cross-neutralization potency against different genotypes of PEDV, and the IM group is superior to the IN group regarding NAb titers. Furthermore, the twice-immunized rAd5-PEDV-S IM group was higher than the once-immunized IM group (administered at 5 weeks before farrowing), and no significant differences were found between the twice-immunized rAd5-PEDV-S IM group and the once-immunized IM group (administered 5 weeks before farrowing), indicating their effectiveness in inducing a NAb response.

### rAd5-PEDV-S protects piglets against PEDV challenge

**Fig 3:**
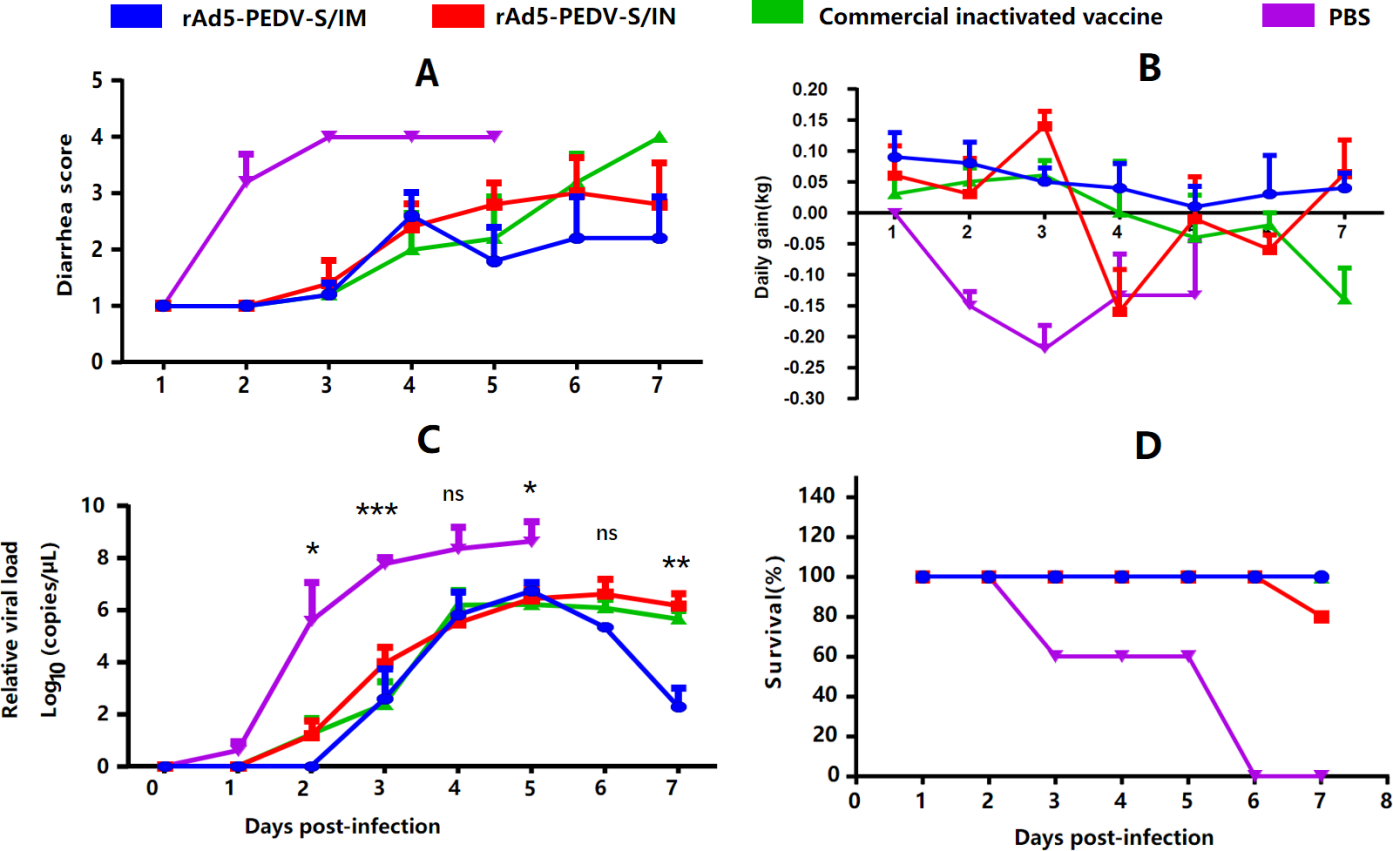
Clinical symptom of PED-infected piglets. (**A**) Diarrhea scores of PED-infected piglets. (**B**) Daily gain of PED-infected piglets. (**C**) PEDV genomic RNA copies of PED-infected piglets. (**D**) Survival rate of PED-infected piglets. (*, *P* < 0.05; **, *P* < 0.01; ***, *P* < 0.001; ****, *P* < 0.0001; ns, no significant).

**Fig 4:**
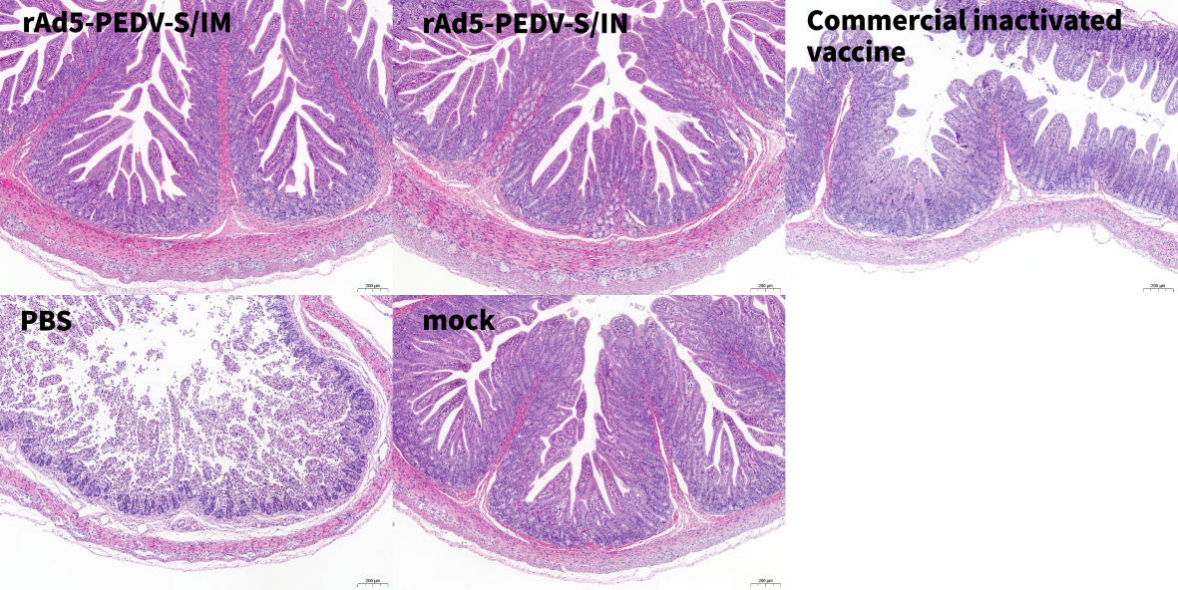
Pathological analysis of PED-infected piglets. The ileum of each pig was collected and stained with hematoxylin and eosin and examined using light microscopy.

To measure the protection afforded by rAd5-PEDV-S in sows, 5-day-old suckling piglets born to the rAd5-PEDV-S immune sows and other groups were selected for a PEDV challenge. All piglets challenged with the PEDV-KB4 strain exhibited classic PED symptoms, including diarrhea. However, the severity of diarrhea was milder in the rAd5-PEDV-S IM group (scores <3) and improved by 5 days post-infection (dpi) (Fig. 3A). In contrast, piglets in the PBS group displayed severe diarrhea (scores 3–4) (Fig. 3A). The daily weight gain in the rAd5-PEDV-S IM group was relatively stable (+0.049 kg/day) than piglets in the other groups. Piglets in the PBS group exhibited the most significant decrease (–0.159 kg/day) in daily weight gain (Fig. 3B). To evaluate PEDV replication in the feces of challenged piglets, the quantitative reverse transcription polymerase chain reaction (qRT-PCR) method was used to quantify PEDV load. The viral loads in samples collected from the rAd5-PEDV-S IM group were significantly lower than in the PBS group (*P* < 0.05) (Fig. 3C). Notably, viral loads began to decrease in the rAd5-PEDV-S IM group at 6 dpi (10^5.35 ± 0.55^ copies/g). They were significantly lower than that in the commercial inactivated vaccine group at 7 dpi (10^2.30 ± 2.67^ copies/g) (Fig. 3C). The survival rate of piglets in the rAd5-PEDV-S IM group and the commercial inactivated vaccine group was 100% (5/5). In contrast, the survival rate was 80% (4/5) in the IN group and 0% (0/5) in the PBS group (Fig. 3D). The histopathological changes in the small intestine of PEDV-infected piglets were investigated using hematoxylin and eosin staining. Mucosal epithelial cell shedding and inflammatory cell infiltration were observed in some ileum tissues of the piglets (Fig. 4). These changes were most visible and evident in the PBS control group, where the mucosal layer showed substantial mucosal epithelial cell shedding and intestinal villous shedding. In contrast, the other groups showed no evident pathological changes (Fig. 4). These findings suggest that rAd5-PEDV-S can reduce PEDV proliferation and alleviate the pathology in small intestinal tissues in PEDV-infected piglets. The piglets produced by rAd5-PEDV-S IM sows resisted PEDV infection, had fewer clinical symptoms, and demonstrated less intestine pathological damage.

## DISCUSSION

PED is a considerable threat to the swine industry, and vaccination is the most efficient way to prevent the disease (23, 25); however, the emergence of novel G2b PEDV variants has led to vaccine failure, hindered the prevention and control of PED, and caused substantial economic losses in China (3, 26 – 28). The replication of novel PEDV variants *in vitro* is another substantial challenge, resulting in difficulty in isolating novel strains (29 – 31). The S protein is the structural protein of the virus, containing a receptor binding domain that mediates viral invasion of host cells and an antigenic epitope that mediates the organism’s production of NAb (32, 33). The novel adenovirus vector vaccine based on the S gene is a suitable strategy to prevent coronavirus-related diseases, especially for the virus variant such as SARS-COV-2 (9). This study first constructed a candidate PED vaccine expressing the S protein of G2b PEDV based on the Ad5 system.

Many studies found that PEDV-specific IgA and IgG antibodies in serum and colostrum from late-term pregnant sows after PEDV vaccination protect piglets from PEDV infection, as passive lactation immunization is a promising way to protect newborn piglets from PED (34 – 36). PEDV-exposed gilts can passively transfer maternal immunity via the gut-mammary-gland-secretory IgA axis and protect PED after challenge (28, 36). This study evaluated humoral immunity to the rAd5-PEDV-S vaccine and compared the effectiveness of the IM and IN immunization routes. These procedures allow for assessing the immunogenicity and efficacy of the rAd5-PEDV-S vaccine in generating an immune response in sows and evaluating its potential use against PEDV infection. We found that rAd5-PEDV-S induced high levels of colostrum IgA and serum IgG titers in sows; the antibody levels in the IM were higher than that in the other groups (*P* < 0.05) (Fig. 2B and C). The rAd5-PEDV-S administered via the IM route was more effective than the IN route. Other studies confirmed that the PED recombinant adenovirus vector vaccine induced the production of IgA and IgG antibodies (20, 21).

NAb is used to measure an individual’s immune status to control infectious diseases and is often an indicator associated with vaccine protection (37). We chose the G2a PEDV strain KB4 and the G1b PEDV strain CV777 to determine the titers of NAb. The results showed that the NAb titers of rAd5-PEDV-S were higher than the commercial inactivated vaccine, and the rAd5-PEDV-S IM route was higher than IN (*P* < 0.05) (Fig. 2D and E). A previous study demonstrated that neutralizing antibodies induced by rAd-heat-labile enterotoxin B (LTB)-core neutralizing epitope (COE) in BALB/c mice were effective against the G1 PEDV strain SM98 and the G2 strain HID9051, with mean titers of 1:32 and 1:8, respectively (21). NAb induced by PED recombinant adenovirus in 4-week-old pigs was 1:64 (20). These findings suggest that the rAd5-PEDV-S vaccine induces cross-neutralizing titers in pregnant sows against two different genotypes of PEDV, with high titers being recorded, which was superior to findings in previous studies (20, 21). These findings have critical implications for developing effective vaccines against PEDV infection in swine.

Interestingly, the twice-immunized rAd5-PEDV-S IM group was higher than the once-immunized IM group (administered at 5 weeks before farrowing), but there was no significant difference in IgA, IgG, and NAb titers between the twice-immunized rAd5-PEDV-S IM group and the once-immunized IM group (administered at 5 weeks before farrowing) (*P* > 0.05), which may be due to the preexisting anti-adenovirus antibody in host primarily immunized by rAd5-PEDV-S (38). This finding suggests that administering the single dose of rAd5-PEDV-S vaccine to sows at 5 weeks before farrowing could also be a viable option. Currently, most commercial vaccines for PEDV involve a twice immunization program, where sows were immunized separately at 5 and 2 weeks before farrowing (15). However, the rAd5-PEDV-S vaccine exhibited excellent immunogenicity with a single dose administered 5 weeks before farrowing. This result was consistent with another study on the adenovirus vector vaccine; only one dose induced an excellent immune response (9). This finding has implications for reducing problems such as stress in sows due to the multiple immunizations required with traditional vaccines.

Virus challenge testing with the live virus is essential to evaluate the protective nature of the vaccine. PED infection mainly causes severe diarrhea and small intestine damage (1). Although there was no significant difference in antibody titers between the twice-immunized rAd5-PEDV-S IM group and the once rAd5-PEDV-S IM group (administered 5 weeks before farrowing), the antibody titers in the twice-immunized IM group were higher (*P* > 0.05) (Fig. 2). Only piglets produced by the twice immunization group were selected. Suckling piglets in each immunization group were challenged with the PEDV-KB4 strain. The piglets in the PBS group developed diarrhea and weight loss (Fig. 3). These findings were consistent with a previous study in pigs, highlighting the pathogenic nature of the virus and its effect on the digestive system (39). However, the piglets from candidate vaccine rAd5-PEDV-S groups improved the clinical symptoms of diarrhea and alleviated the impact on the weights of the piglets. PEDV RNA expression in the feces of the PEDV-challenged PBS group was significantly higher than in the other groups, and low-level RNA expression was detected in rAd5-PEDV-S IM group piglets. The rAd5-PEDV-S IM antibody produced after immunization provided 100% protection in piglets and 80% protection in the IN group, indicating that the antibody produced after immunization with rAd5-PEDV-S protected PEDV-infected piglets (Fig. 3).

Similarly, the small intestine pathological analysis suggested that the rAd5-PEDV-S-immunized groups showed mild histopathological changes and only a small amount of inflammatory cell infiltration in the ileum. In contrast, the histopathological findings of the ileum in the PBS group showed that the mucosal layer showed a large amount of mucosal epithelial cell shedding, intestinal villi shedding, and inflammatory cell infiltration, which were consistent with pathological changes in PED (11, 39) (Fig. 4). These results demonstrate that immunization with the recombinant vaccine candidate strains induces protection against a PEDV challenge. The protective effect was superior to previous studies from the analysis (20).

In conclusion, we constructed a PED candidate vaccine rAd5-PEDV-S, and the humoral immunity of sows immunized with rAd5-PEDV-S was evaluated in this study. The results showed that the sows immunized with rAd5-PEDV-S could induce an excellent humoral immune response against PEDV. The piglets produced by rAd5-PEDV-S IM sows improved diarrhea after a challenge and reduced intestine pathological damage. This study provides a basis for developing PEDV vaccines.

## MATERIALS AND METHODS

### Plasmids, viruses, and cells

The highly virulent Chinese G2b PEDV strain CH/TP-4-4/2018 (GenBank accession number: MK140814.1) was isolated by our laboratory. HEK293 cells and AdMax adenovirus system (pDC316 and pBHGloxΔE1, 3Cre) derived from Ad5 were obtained from Microbix Biosystems Inc. (Canada). Vero cells were kept in our laboratory. HEK293 and Vero cells were grown in Dulbecco’s Modified Eagle Medium (Invitrogen, USA) containing 10% heat-inactivated fetal bovine serum (Invitrogen, Australia) and 1% antibiotics and antimycotics (10,000 units of penicillin, 10,000 µg of streptomycin, and 25 µg of Fungizone per milliliter) (Gibco, USA) and cultured at 37°C with 5% CO_2_.

### Construction of recombinant adenovirus expressing PEDV S protein

The full S protein gene of PEDV based on the CH/TP-4-4/2018 strain (MK140814.1) was codon optimized by Songon Biotech (Shanghai, China) for increased expression in mammalian cells. The gene was synthesized with *EcoR*I and *Hind*III upstream and downstream of the open reading frame, respectively, and cloned into the shuttle plasmid of the AdMax adenovirus system (Microbix Biosystem, Canada) by enzyme digestion and ligation. After sequencing identification, the shuttle plasmid with the target gene was co-transfected into HEK293 cells with the backbone plasmid (pBHGloxΔE1, 3Cre) by jetPRIME transfection reagent (POLYPLUS, France) according to the manufacturer’s instructions. The transfected cells were passaged when overgrown and collected until Ad-related cytopathic effects were observed. The cells were lysed by three freeze-thaw cycles to release the recombinant viruses.

PCR and double digestion identified the recombinant vector. The recombinant virus (rAd5-PEDV-S) was serially passaged to 15 passages, and primary, 5, 10, and 15 passages were used to verify whether the plasmid could exist stably. The universal primers of pDC316 were used to amplify the S gene.

HEK293 cells infected with rAd5-PEDV-S at a multiplicity of infection (MOI) = 0.1 were collected and lysed using a radioimmunoprecipitation assay buffer (RIPA) protein extraction reagent (Thermo Scientific, USA) for each well at 48 h post-infection. The cell lysate was centrifuged, and the supernatant was collected, mixed with loading buffer (Thermo Scientific, USA), and run on SDS-PAGE. A BIO-RAD transfer system transferred protein to a polyvinylidene fluoride (PVDF) membrane (BIO-RAD, USA). Membranes were blocked for 1 h in 5% skim milk with 0.1% Tween-20. A monoclonal mouse anti-PEDV S antibody (1:1,000 dilution) was added and incubated for 2 h at 37°C as the primary antibody. Next, the horseradish peroxidase (HRP)-conjugated goat anti-mouse IgG antibody (Abcam, UK, 1:10,000 dilution) was added and incubated for 2 h at 37°C as the secondary antibody. As an internal parameter, β-actin was detected on the same membrane by an HRP-conjugated anti-β-actin antibody (Abcam, UK, 1:10,000 dilution). Protein bands were processed using an enhanced chemiluminescence (ECL) detection kit (Beyotime Biotechnology, China) and visualized using a chemiluminescence image analysis system (BIO-RAD, USA).

IFA was used to verify the expression of the S protein. HEK293 cells were infected with rAd5-PEDV-S at an MOI = 0.1. A monoclonal mouse anti-PEDV S antibody was added and incubated as the primary antibody. Next, the fluorescein isothiocyanate (FITC)-conjugated goat anti-mouse IgG antibody (Abcam, UK, 1:100 dilution) was added and incubated as the secondary antibody. Observe the periphery of the cell nucleus under a fluorescence microscope for the appearance of green fluorescence.

### Chromatographic purification of rAd5-PEDV-S

The rAd5-PEDV-S was purified using Diamond Layer 700 BA, which involves anionic and hydrophobic interactions and molecular sieving, and Diamond Q Mustang, which involves ion exchange interactions. The process included primary purification (10,000 rpm/min, centrifugation at 4°C for 10 min; 0.2 µM filter), exhaust (flush the pipe with ddH_2_O, 20 mL/min, and flush both A and B pipes, until the conductivity and absorbance curves were balanced), equilibration [wash the chromatographic column with balance buffer (PBS, pH7.5) until the pH and conductivity of the buffer at the outlet were the same as that of the balance buffer, which usually requires three to five column bed volumes], loading (add the sample to the purification column containing the medium, the flow rate was 0.6 mL/min), cleaning [wash the chromatographic column with equilibrium buffer (PBS, pH7.5) to elute the impurities retained on the medium until the UV absorption was close to the baseline], and re-equilibration [impurities bound on the medium need to be eluted under strong elution conditions (1M NaOH +1M NaCl)]. The total VP was quantified using an ultraviolet spectrophotometer analysis, where one OD260 corresponded to ~1.1 × 10^12^ VP. The purity of the batch was considered adequate if the A260/A280 ratio was between 1.2 and 1.3 based on qualitative analysis. To verify the stability of the plasmid, the purified rAd5-PEDV-S was infected into HEK293 cells. The S gene was amplified with universal primers of pDC316, and the expression of the S protein was verified using western blotting. After purification, the TCID_50_ of the purified rAd5-PEDV-S was calculated using the Reed-Muench method.

### Sow vaccination

Forty commercial sows were randomly divided into eight groups of five each (Table S5). Before the study, virus-specific PCR collected fecal swabs from sows and tested them for PEDV, transmissible gastroenteritis virus (TGEV), porcine deltacoronavirus (PDCoV), and porcine rotaviruses. The results of the PCR tests were negative for all tested viruses in the fecal swabs collected from the sows. We compared different immune routes and times to evaluate the best immunization program for rAd5-PEDV-S. The vaccine route of IM was post-auricular neck muscle injection 2 mL (2 × 10^9^ TCID_50_), and the mode of IN was transnasal instillation 2 mL (2 × 10^9^ TCID_50_). Immune times include sows being immunized with a primary immunization at 5 weeks before farrowing and a secondary immunization with the same dose irradiated 2 weeks before farrowing and immunized only once each at 5 weeks before or 2 weeks before farrowing. The sows were observed daily for mental status, appetite, and body temperature.

### Indirect ELISA assay

For the PEDV-specific IgG and IgA assays in sows, 96-well polystyrene high-binding microplates (NEST, China) were utilized as the assay platform. The microplates were coated with inactivated PEDV (KB4 strain, TCID_50_ = 1 × 10^6^) and then incubated overnight at 4°C. The plates were blocked with 5% skim milk. Next, a series of dilutions of serum samples were added to the plate wells for binding (diluted in eight gradients, and diluted 10 times between each gradient). The microplates were incubated with 100 µL of 1:3,000 diluted anti-pig IgG antibodies (Abcam, UK) or anti-pig IgA antibodies (Abcam, UK), conjugated with HRP. The endpoint titer for each serum sample was defined as the highest reciprocal serum dilution that yielded an absorbance ≥2.1-fold over the negative control serum values, indicating the presence of PEDV-specific IgG or IgA antibodies in the serum sample.

### PEDV neutralization assay

The PEDV neutralization assay in serum and colostrum was conducted to determine the NAb titer against the CV777 or KB4 strains. The NAb assay was performed in Vero cells (2 × 10^5^ cells/well) with serum or colostrum samples (diluted in eight gradients, and diluted two times between each gradient) in triplicate per dilution; 100 µL of the diluted samples were mixed with an equal volume of CV777 or KB4 virus supernatant containing 200 TCID_50_/0.1 mL and then incubated at 37°C incubator for 96 h. This assay involves observing the CPE of Vero cells and quantifying the degree of neutralization present in serum or colostrum samples using the Reed-Muench method, which will indicate potential immunity to PEDV infection.

### PEDV challenge of suckling piglets born to the immune sows

A PEDV viral challenge was performed to evaluate the protective effect of the rAd5-PEDV-S vaccine in piglets born to immune sows. Five-day-old piglets born to immune sows were randomly selected to receive 1 mL of PEDV viral fluid (KB4 strain, 10^6^ TCID_50_) orally, and the piglets in the MOCK group were not subjected to any operation. Daily observations and recordings were made of piglet mental status, feed intake, body weight, temperature, and fecal collection after the challenge. Clinical symptoms were displayed in Table S6. Rectal swabs were collected for qRT-PCR (EXONGEN, China) to detect sub-genomic RNA. The method of detection and optimization referred to extracting RNA and reverse transcribing cDNA from equal weights of minipig feces (39). A histopathological examination was performed following a previously established protocol to investigate the effects of the rAd5-PEDV-S vaccine on PEDV infection in piglets (40). Briefly, the ileum was collected and fixed with 4% paraformaldehyde at room temperature for 48 h, then embedded in paraffin. The tissue samples were sliced into 5 µm sections, stained with hematoxylin and eosin, and examined using light microscopy. These tests and observations allowed for monitoring of the health and immune response of piglets after the viral challenge and the effectiveness of the rAd5-PEDV-S vaccine produced by the immune sows in conferring immunity to their offspring.

### Statistical analysis

The GraphPad Prism version 8 software package (Prism) was used for the data analysis. Firstly, we analyzed variance (ANOVA) to evaluate the statistical differences between different groups. The ANOVA analysis results confirmed significant differences between the groups. To further compare the mean differences between groups, we used Tukey’s multiple comparison test for subsequent analysis. Significance was denoted as follows: *, *P* < 0.05; **, *P* < 0.01; ***, *P* < 0.001; ****, *P* < 0.0001.

## AFTER EPUB

*[This article was published on 4 December 2023 with incorrect and incomplete information in the funding table. The funding table was updated in the current version, posted on 15 December 2023.]*

## SUPPLEMENTAL MATERIAL

Fig. S1 spectrum.02403-23-s0001.tiff Clinical symptom vaccinated piglets.

Supplemental tables spectrum.02403-23-s0002.docx Tables S1 to S6; legend to Fig. S1.

ASM does not own the copyrights to Supplemental Material that may be linked to, or accessed through, an article. The authors have granted ASM a non-exclusive, world-wide license to publish the Supplemental Material files. Please contact the corresponding author directly for reuse.
