# Supplementary figures and images for: Immunogenicity and protective efficacy of recombinant adenovirus expressing a novel genotype G2b PEDV spike protein in protecting newborn piglets against PEDV
Source: Microbiol Spectr. 2023 Dec 4;12(1):e02403-23. doi: 10.1128/spectrum.02403-23 (PMC10783080; doi:10.1128/spectrum.02403-23)

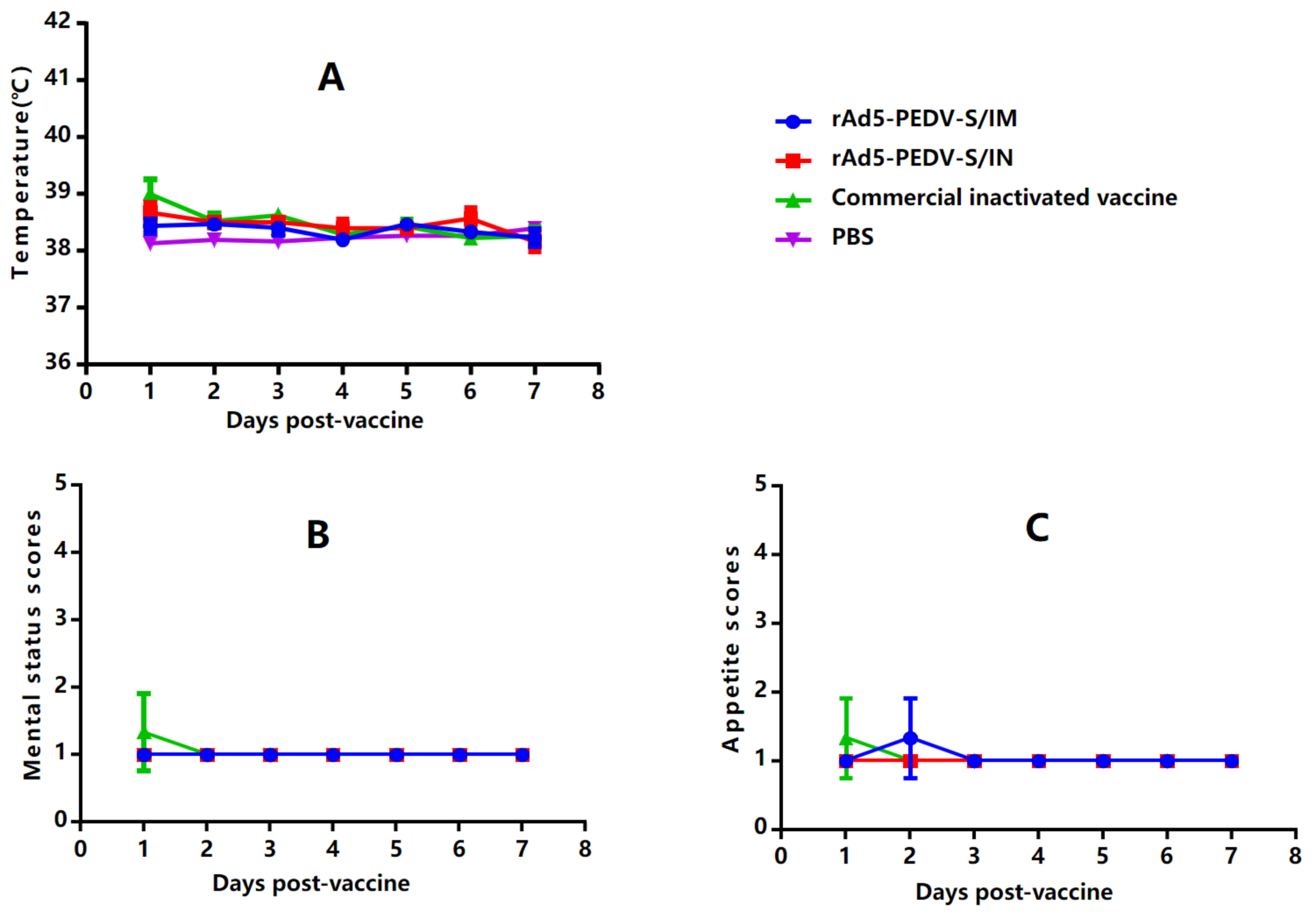

Supplement: Fig. S1 — Clinical symptom vaccinated piglets. [file spectrum.02403-23-s0001.tiff]
