# Supplementary material for: Immunogenicity and protective efficacy of recombinant adenovirus expressing a novel genotype G2b PEDV spike protein in protecting newborn piglets against PEDV
Source: Microbiol Spectr. 2023 Dec 4;12(1):e02403-23. doi: 10.1128/spectrum.02403-23 (PMC10783080; doi:10.1128/spectrum.02403-23)
Supplement: Supplemental tables — Tables S1 to S6; legend to Fig. S1. [file spectrum.02403-23-s0002.docx]

**Supplementary**

**Table S1. The IgA titers of colostrum.**

| Group | IgA titers |
| --- | --- |
| rAd5-PEDV-S/IM/2doses | 1:10^4.0±0^ |
| rAd5-PEDV-S/IN/2doses | 1:10^2.3±0.7^ |
| Commercial inactivated vaccine | 1:10^2.3±0.7^ |
| rAd5-PEDV-S/IM/1dose/5w | 1:10^3.3±0.7^ |
| rAd5-PEDV-S/IN/1dose/5w | 1:10^2.6±0.6^ |
| rAd5-PEDV-S/IM/1dose/2w | 1:10^3.3±0.7^ |
| rAd5-PEDV-S/IN/1dose/2w | 1:10^2.6±0.6^ |

**Table S2. The IgG titers of serum.**

| Group | IgG titers |
| --- | --- |
| rAd5-PEDV-S/IM/2doses | 1:10^4.3±0.7^ |
| rAd5-PEDV-S/IN/2doses | 1:10^4.0±0^ |
| Commercial inactivated vaccine | 1:10^3.6±0.6^ |
| rAd5-PEDV-S/IM/1dose/5w | 1:10^3.6±0.6^ |
| rAd5-PEDV-S/IN/1dose/5w | 1:10^2.3±0.7^ |
| rAd5-PEDV-S/IM/1dose/2w | 1:10^4.3±0.7^ |
| rAd5-PEDV-S/IN/1dose/2w | 1:10^2.6±0.6^ |

**Table S3. The neutralizing antibody titers of colostrum.**

| Group | KB4/G2a | CV777/G1b |
| --- | --- | --- |
| rAd5-PEDV-S/IM/2doses | 1:748±426 | 1:404±184 |
| rAd5-PEDV-S/IN/2doses | 1:310±44 | 1:52±37 |
| Commercial inactivated vaccine | 1:118±29 | 1:154±54 |
| PBS | 1:28±12 | 1:22±6 |
| rAd5-PEDV-S/IM/1dose/5w | 1:505±90 | 1:197±98 |
| rAd5-PEDV-S/IN/1dose/5w | 1:135±42 | 1:44±22 |
| rAd5-PEDV-S/IM/1dose/2w | 1:105±22 | 1:38±21 |
| rAd5-PEDV-S/IN/1dose/2w | 1:32±16 | 1:54±8 |

**Table S4. The neutralizing antibody titers of serum.**

| Group | KB4/G2a | CV777/G1b |
| --- | --- | --- |
| rAd5-PEDV-S/IM/2doses | 1:315±39 | 1:216±83 |
| rAd5-PEDV-S/IN/2doses | 1:36±38 | 1:52±37 |
| Commercial inactivated vaccine | 1:22±4 | 1:50±24 |
| PBS | <1:16±0 | <1:16±0 |
| rAd5-PEDV-S/IM/1dose/5w | 1:314±20 | 1:114±63 |
| rAd5-PEDV-S/IN/1dose/5w | 1:48±22 | 1:22±2 |
| rAd5-PEDV-S/IM/1dose/2w | 1:127±23 | 1:49±27 |
| rAd5-PEDV-S/IN/1dose/2w | 1:51±19 | 1:37±16 |

**Table S5. Immune program in this study**

| Vaccine | Immune route | Immune dose | |
| --- | --- | --- | --- |
|  |  | 5 weeks before farrowing | 2 weeks before farrowing |
| rAd5-PEDV-S | IM | 2×10^9^ TCID_50_ (2mL) | 2×10^9^ TCID_50_ (2mL) |
| rAd5-PEDV-S | IN | 2×10^9^ TCID_50_ (2mL) | 2×10^9^ TCID_50_ (2mL) |
| Commercial inactivated vaccine | IM | 2mL | 2mL |
| PBS | IM | 2mL | 2mL |
| rAd5-PEDV-S | IM | 2×10^9^ TCID_50_(2mL) | / |
| rAd5-PEDV-S | IN | 2×10^9^ TCID_50_(2mL) | / |
| rAd5-PEDV-S | IM | / | 2×10^9^ TCID_50_(2mL) |
| rAd5-PEDV-S | IN | / | 2×10^9^ TCID_50_(2mL) |
|  |  |  |  |

**Table S6.** **The clinical symptom scores of piglets.**

| Items | Evaluation criterion | Score |
| --- | --- | --- |
| Diarrhea | normal | 1 |
|  | Fecal soft | 2 |
|  | Mild diarrhea | 3 |
|  | Severe watery diarrhea | 4 |
| Appetite | Normal | 1 |
|  | Reluctant to move, nonviable | 2 |
|  | Poor appetite | 3 |
|  | Stop eating | 4 |
| Mental status | Normal | 1 |
|  | Be listless | 2 |
|  | Lay frequently on the stomach and stood occasionally | 3 |
|  | Be at one's last gasp | 4 |

Figure and Table legends

Fig S1. Clinical symptom vaccinated piglets. (A) The rectal temperature of sows post vaccination; (B) The mental status scores of sows post vaccination; (C) The appetite scores of sows post vaccination.

Table S1. The IgA titers of colostrum.

Table S2. The IgG titers of serum.

Table S3. The neutralizing antibody titers of colostrum.

Table S4. The neutralizing antibody titers of serum.

Table S5. Immune program in this study.

Table S6. The clinical symptom scores of piglets
